# Nasal Drug Delivery and Nose-to-Brain Delivery Technology Development Status and Trend Analysis: Based on Questionnaire Survey and Patent Analysis

**DOI:** 10.3390/pharmaceutics16070929

**Published:** 2024-07-11

**Authors:** Yuanyuan Ge, Xingying Xu, Meng Cao, Baijun Liu, Ying Wang, Ping Liao, Jiajing Wang, Yifei Chen, Hongmei Yuan, Guiliang Chen

**Affiliations:** 1 School of Business Administration, Shenyang Pharmaceutical University, Shenyang 110016, China; gemiracle@163.com (Y.G.); lbj1003@foxmail.com (B.L.); 15383267523@163.com (Y.W.); 2 Shanghai Center for Drug Evaluation and Inspection, Shanghai 201203, China; caomeng@smda.sh.cn (M.C.); liaoping@smda.sh.cn (P.L.); wangjiajing@smda.sh.cn (J.W.); chenyifei25@smda.sh.cn (Y.C.); 3 Shanghai Library (Institute of Scientific and Technical Information of Shanghai), Shanghai 200031, China; xyxu@libnet.sh.cn

**Keywords:** nasal drug delivery, nose-to-brain delivery(N2BD), questionnaire survey, patent analysis, LDA topic model

## Abstract

Nasal administration is a non-invasive method of drug delivery that offers several advantages, including rapid onset of action, ease of use, no first-pass effect, and fewer side effects. On this basis, nose-to-brain delivery technology offers a new method for drug delivery to the brain and central nervous system, which has attracted widespread attention. In this paper, the development status and trends of nasal drug delivery and nose-to-brain delivery technology are deeply analyzed through multiple dimensions: literature research, questionnaire surveys, and patent analysis. First, FDA-approved nasal formulations for nose-to-brain delivery were combed. Second, we collected a large amount of relevant information about nasal drug delivery through a questionnaire survey of 165 pharmaceutical industry practitioners in 28 provinces and 161 different organizations in China. Third, and most importantly, we conducted a patent analysis of approximately 700+ patents related to nose-to-brain delivery, both domestically and internationally. This analysis was conducted in terms of patent application trends, technology life cycle, technology composition, and technology evolution. The LDA topic model was employed to identify technological topics in each time window (1990–2023), and the five key major evolution paths were extracted. The research results in this paper will provide useful references for relevant researchers and enterprises in the pharmaceutical industry, promoting the further development and application of nasal drug delivery and nose-to-brain delivery technology.

## 1. Introduction

The administration of drugs through the nasal cavity, also known as nasal drug delivery (intranasal delivery), has gained significant attention in recent years due to its potential for efficient and rapid drug absorption. The nasal route offers several advantages over traditional oral and parenteral drug delivery methods, including the avoidance of first-pass metabolism, rapid onset of action, and non-invasive administration [1,2,3,4,5,6]. These unique characteristics make nasal drug delivery an attractive option for both systemic and local drug delivery [7,8,9].

**Figure 1:**
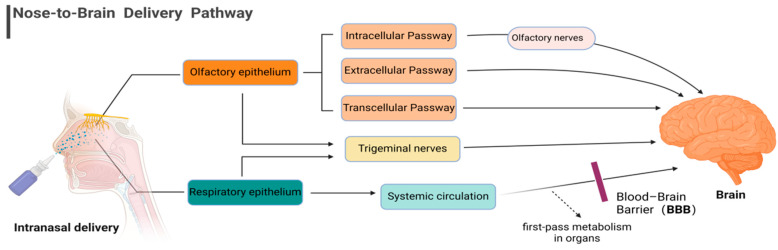
Main nose-to-brain delivery pathway.

One of the most promising applications of nasal drug delivery is in the field of nose-to-brain delivery (N2BD). This route allows drugs to bypass the blood–brain barrier (BBB) and directly target the central nervous system (CNS), offering a potential solution for the treatment of neurological disorders [10,11,12,13,14,15,16]. Several studies have detailed the transportation of drugs to the brain via the nasal route [17,18,19,20]. It is widely agreed that drug transfer from the nose to the brain mainly occurs in the respiratory and olfactory regions of the nasal cavity, with the olfactory region serving as the primary pathway [16]. Figure 1 illustrates the main N2BD pathway.

Currently, there are several nasal drug delivery products on the market, such as nasal sprays, nasal drops, and nasal powders. These products are used for the treatment of various conditions, such as allergies, nasal congestion, and migraines. However, challenges remain in the development of N2BD systems, including the need for improved drug targeting, drug stability, and safety profiles [2,3,4].

In this study, we aim to analyze the current status and trends in nasal drug delivery and N2BD technologies based on a survey of researchers and an analysis of patents. By understanding the advantages, disadvantages, and challenges of these technologies, we hope to provide insights into the development of novel drug delivery systems and the treatment of neurological disorders.

## 2. Materials and Methods

### 2.1. Literature Research and Market Analysis

In this paper, literature research and market analysis were conducted to understand the current development status of nasal drug delivery formulations and the basic situation of marketed and investigational products.

We searched the relevant drug databases of the US FDA, EU EMA, Japan PMDA, and China NMPA to learn about the approved and marketed nasal drug delivery products, and we focused on analyzing the US FDA-approved nasal formulations targeting the N2BD pathway.

### 2.2. Questionnaire Survey

The survey was designed by the research authors and then outsourced to a third-party organization (OurYao.com) for distribution through “Questionnaire Star”. It comprised 24 questions, including single-choice, multiple-choice, and fill-in-the-blank queries. These questions covered participant demographics, enterprise and product types, interest in nasal drug delivery products, ongoing product development activities, N2BD technology, and challenges in developing nasal drug delivery products. The survey was conducted online in an open-ended format. Targeting professionals in the drug-related industry in China, the survey utilized a web-based distribution method. It was available from 21 February to 6 March 2023, resulting in 169 electronic responses. After removing 4 duplicates, 165 valid responses remained, originating from 28 provinces and 161 different units in China.

### 2.3. Patent Analysis

#### 2.3.1. Data Sources and Search Methods

The patent data used in this study primarily originated from the Derwent Innovation (DI) database by Clarivate Analytics, a globally recognized consultancy, and the IncoPat global patent database. In this research, IncoPat and Derwent Innovation served as the primary analytical instruments for assessing and illustrating nose-to-brain delivery patents, supported by IncoPat’s data analysis platform, DI, Python, and data processing tools such as Excel, aiming to provide a comprehensive overview of the landscape of nose-to-brain drug delivery technologies.

The search formula for this study was (TIAB = (鼻 OR nasal OR nose) AND TIAB = (脑 OR 颅 OR 中枢神经 OR brain OR encephalon OR harns OR pericranium OR cerebrum OR cranial OR “central nervous” OR CNS) AND TIAB = (靶向 or 给药 or 递送 or target* or delivery)), and the patent type was limited to patents for inventions. The search date was 31 December 2023, and after data cleaning by homologous merging, removing duplicates, and manual screening, 704 global patent application literature datasets for N2BD technology were obtained and used for further analysis.

#### 2.3.2. Topic Evolution Analysis Based on the LDA Model

Patent text mining can discover potential data patterns and internal relations from a large amount of unstructured textual information and is an important method for technical topic evolution analysis. In this study, we use the Latent Dirichlet Allocation (LDA) model to identify the technical topics contained in patents, as a part of the technical topic evolution analysis. Technological theme evolution refers to the dynamic change of technological theme in a continuous, sliding time window, describes the vein of technological innovation development over time, and analyzes the law of technological innovation from a dynamic perspective. The LDA model was proposed by Blei [21], a kind of unsupervised machine learning model that extracts and analyzes the topics from short text at the probabilistic statistical level. It is a three-layer Bayesian model constructed by “document–topic–word”, which can be used to mine large-scale text data. At present, the research methods of technological topic evolution analysis mainly fall into three categories:

(a) Based on the bibliometric method, the present situation and trend of technology development are studied through the introduction of quantitative indicators and the analysis of quantitative trends. Zhang [22] summarized the evolution of virtual reality technology by using keyword frequency. Yoon [23] used patent technology classification to analyze technology evolution. Although this method can judge the trend of technology development, the description of the evolution of patent technology is too rough to make in-depth use of the large amount of useful information contained in the patent text and, therefore, cannot meet the need to explore the direction of technology evolution in a specific field.
(b) Based on the patent citation analysis method, the flow of technological innovation is reflected through the directed citation relationship between patents. Verspagen [24] constructed a fuel cell patent citation network to extract the technology evolution path. Huang [25] applied the co-classification analysis of patent citations to the field of 3D printing to reveal the technology hotspots and technology development track. Although the method based on patent citation analysis has high accuracy, it has the disadvantage of time lag. Some patents that are cited less frequently but contain important technical information are easy to be ignored, resulting in inaccurate results.
(c) Based on the text mining method, the evolution analysis was carried out by extracting technical information from unstructured text data, such as patent title and abstract. Zhu [26] used the Latent Dirichlet Allocation topic model to extract the subject words of patent text, extract the technical nodes whose text similarity exceeded the threshold, and generate the technical evolution path. Hu [27] used the LDA model to extract the technical themes of the new energy automobile industry and show the development trend of industrial technology. The research on this kind of method has gradually formed a research trend that focuses on the application of LDA and its extended model, supplemented by other text mining technologies, such as word embedding and deep learning. This kind of method is often combined with the first two kinds of methods and has attracted wide attention.

In summary, the methods based on bibliometric and patent citation analysis have their limitations. As a result, they are not comprehensive and accurate enough to describe the process of topic change in the technical field, and the accuracy and scientificity of the research results need to be improved, while the application of the LDA topic model to study the evolution of technical topics can accurately identify the technical topics. The LDA topic model has been widely used in many fields of topic recognition, and can effectively analyze large-scale unstructured document sets [28,29,30,31].

**Figure 2:**
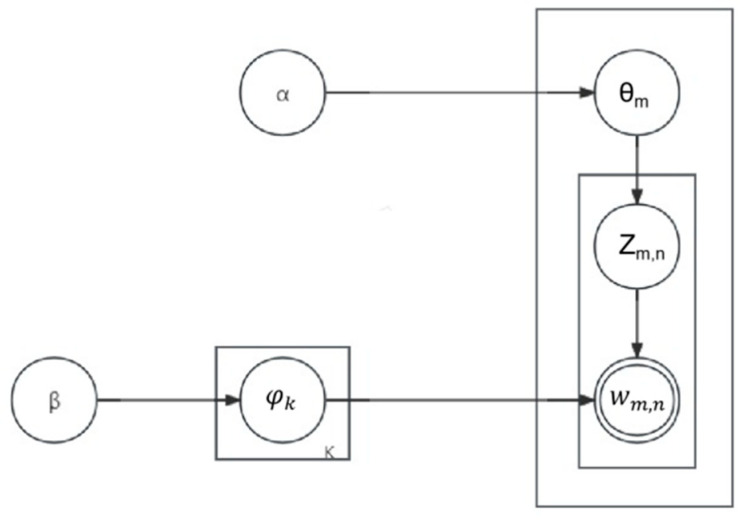
Probabilistic graph model of the Latent Dirichlet Allocation (LDA) algorithm.

Figure 2 shows the LDA model construction process. The LDA model first extracts a topic in each document, then randomly selects a word from the vocabulary corresponding to the extracted topic and repeats the above process until every word in the whole document is traversed, finally generating the document–topic distribution and topic–vocabulary distribution.

However, the topics under each time window generated by the LDA topic model exist independently. To explore the evolutionary relationship between topics, the continuity between topics can be measured by calculating the similarity between the topics of neighboring time windows. The similarity between temporal topics is measured by calculating the cosine similarity between the topics of neighboring time windows, and then the correlation relationship between the topics of neighboring time windows is established. The calculation is shown in Equation (1):

(1) $$S(T_{i}^{t-1},T_{j}^{t})=\frac{\sum_{n_{0}=1}^{N_{0}}p(w_{n_{0}}\left|T_{i}^{t-1})\times p(w_{n_{0}}\left|T_{j}^{t})\right.\right.}{\sqrt{\sum_{n_{0}=1}^{N_{0}}{\left[p(w_{n_{0}}\left|T_{i}^{t-1}\right.\right]}^{2}}\times \sqrt{\sum_{n_{0}=1}^{N_{0}}{\left[p\left(w_{n_{0}}\left|T_{j}^{t}\right.\right)\right]}^{2}}}$$

where $T_{i}^{t-1}$ is the *i*th topic under time window *t* − 1,${\;T}_{j}^{t}$ is the *j*th topic under time window *t*, $p(w_{n_{0}}\left|T_{i}^{t-1})\right.$ and $p\left(w_{n_{0}}|T_{j}^{t}\right)$ are the topic–vocabulary distributions under time windows *t* − 1 and *t,*$\;w_{n_{0}}$ is the *n*0th vocabulary in the topic, and *N*_0_ is the total number of vocabularies contained in the topic. The closer the value of $S(T_{i}^{t-1},{\;T}_{j}^{t})$ is to 1, the more similar the topics are. A threshold, *ε*, is set to remove weak and invalid associations: when $S(T_{i}^{t-1},{\;T}_{j}^{t})\;$≥ *ε*, the current topic is a continuation of the previous topic; if $S\left(T_{i}^{t-1},{\;T}_{j}^{t}\right)\;$< *ε*, the current topic is not related to the previous topic. In this study, the threshold, *ε*, was set to 0.2.

The topic evolution model construction was realized based on Python (version 3.9.0), and the text preprocessing was carried out using the Jieba toolkit.

## 3. Results and Discussions

### 3.1. Market Analysis Results

As of 20 June 2023, there have been 688 nasal formulations available in global markets, including sprays, drops, ointments, gels, and dispersions. Nasal sprays are the most commonly used formulation, accounting for 69.7% of the market, followed by drops at 25.1%.

China, the United States, and Japan are the top three countries in terms of approved nasal products, with China having the highest number of approvals, at 209, followed by the United States with 114 and Japan with 106. Nasal drops are the most commonly used nasal product in China, accounting for 55% of the market, while nasal sprays make up 35%. In the United States, nasal sprays are the most popular, constituting 92.9% of domestic nasal products. Japan has a balanced usage between nasal sprays (48.1%) and drops (44.3%).

Table 1 shows the top 10 approved products, which include corticosteroids and non-corticosteroids, such as mometasone, Seroquel, fluticasone, azelastine, and budesonide. These products are used to treat allergic rhinitis, relieve nasal congestion and swelling, and address other indications, such as analgesic fentanyl and the migraine drug sumatriptan.

Based on the drug registration data and literature from the US FDA, there are currently approximately 15 nasal delivery formulations approved for the N2BD pathway. These formulations are indicated for various conditions, such as migraine, depression, epilepsy, opioid overdose, and dry eye, as presented in Table 2.

### 3.2. Questionnaire Survey Results

**Figure 3:**
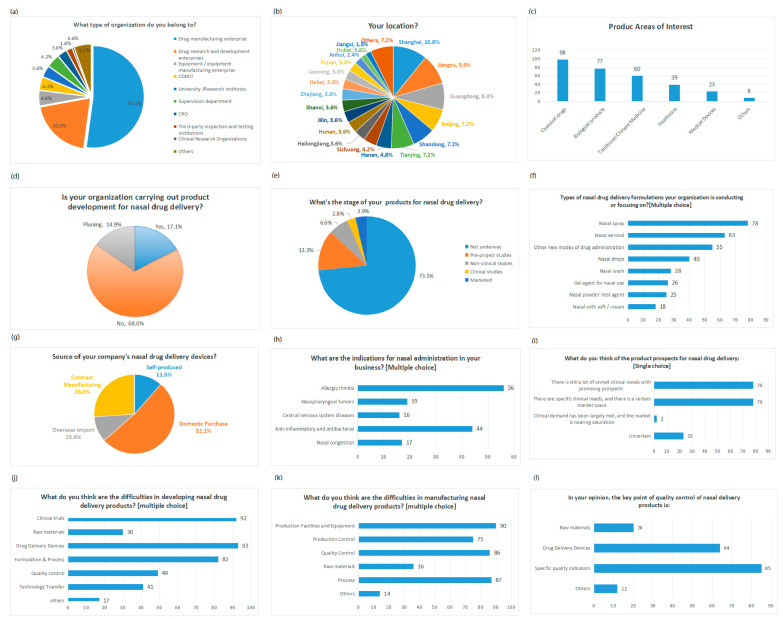
Questionnaire survey results for nasal drug delivery.

Twelve representative questions and responses from the questionnaire are shown in Figure 3.

From the point of view of enterprises in the questionnaire, the 165 respondents in the survey represented 161 different companies. Among them, 86 were pharmaceutical manufacturers, making up 52% of the total, followed by pharmaceutical research and development firms (20%), device manufacturers (5%), and regulatory bodies (4%). Geographically, participants hailed from 28 out of China’s 34 provincial regions. The top six regions were Shanghai, Jiangsu, Guangdong, Shandong, Beijing, and Tianjin, all key players in the pharmaceutical sector, accounting for over 50% collectively. Overall, the participating companies provided insights into the broader landscape of China’s pharmaceutical industry.

From the point of view of the R&D stage of the products, those that have been marketed or are in the clinical research stage accounted for only 7%. This reflects that nasal preparations are still relatively niche projects, and that most enterprises’ projects are still in the stage of consideration or planning. This is small compared to the scale of the entire pharmaceutical industry represented by nasal preparation products.

From the viewpoint of concerned formulation types, device sources, and indications, the most considered dosage forms are nasal sprays, nasal aerosols, and new types of nasal drug delivery. From the viewpoint of device sources, 52.1% of the enterprises’ drug delivery devices are purchased domestically, 10.4% are imported from abroad, 26.0% are commissioned, and 11.5% are self-produced. From the point of view of the indications of the products under development, the most popular indications of the domestic products under development are rhinitis and anti-inflammatory and antibacterial products, and the proportion of those used for central nervous system diseases is 8.84%. It can be seen that the development of domestic nasoencephalic delivery products is still in the initial stage.

From the key points of R&D, production, and quality control of nasal drug delivery, the difficulties in R&D of nasal drug delivery lie in the drug delivery device, clinical trials, and prescription process. The difficulties in production center around the production facilities and equipment, process, and quality control, and the key points of quality control lie in the quality indexes specific to nasal drug delivery, such as the delivered dosage uniformity (DDU), the plume geometry, and the spray pattern.

Furthermore, 43% of the respondents believed that there are significant unmet clinical needs for nasal drug delivery, indicating a promising future. Meanwhile, another 43% believed that there is a specific market space for clinical needs, and only 1% felt that the clinical needs for nasal drug delivery have been mostly met, with the market nearing saturation.

The survey questionnaire also included several open-ended questions. Regarding the safety issues of long-term drug administration, respondents expressed the need to optimize formulation and manufacturing process design, use safer excipients, avoid using excipients with safety risks, optimize the dosage of active drug ingredients and excipients, reduce the impact of dosage and concentration, adopt more suitable drug delivery devices and methods, and conduct preclinical studies, such as toxicology and irritation tests.

In terms of evaluating quantitative N2BD, respondents mentioned various in vitro and in vivo studies. In vitro studies included cell experiments, spray pattern characteristics, 3D model deposition, computer simulation, etc. In vivo studies involved selecting an appropriate biomarker for radiation-targeted development, PK, PD, scoring scale, etc. Animal experiments involved pharmacologic evaluations of safety and efficacy and toxicology testing under carefully controlled conditions [1,10].

In regard to the in vivo and in vitro bioequivalence studies, the respondents indicated that nasal administration is difficult to control in terms of dosage. Additionally, there were significant individual differences and a lack of optimal animal models, making it difficult to demonstrate biological distribution and interactions between different tissue parts or cell types. Furthermore, evaluating local pharmacokinetics is challenging. It is important to note that the delivery device has a significant impact on the quality of studies. For instance, atomizers from various brands and based on different principles exhibit notable variations in the delivery rate and dosage of fine particles.

Respondents expressed their desire for unified standards for nasal administration in China, including testing methods and acceptable limits for DDU, guidelines for nasal administration formulations, product-specific guidance for generic drug product development, and unified device standards across different regulatory regions.

### 3.3. Patent Analysis

#### 3.3.1. Overall Trends

##### Patent Application Trends

**Figure 4:**
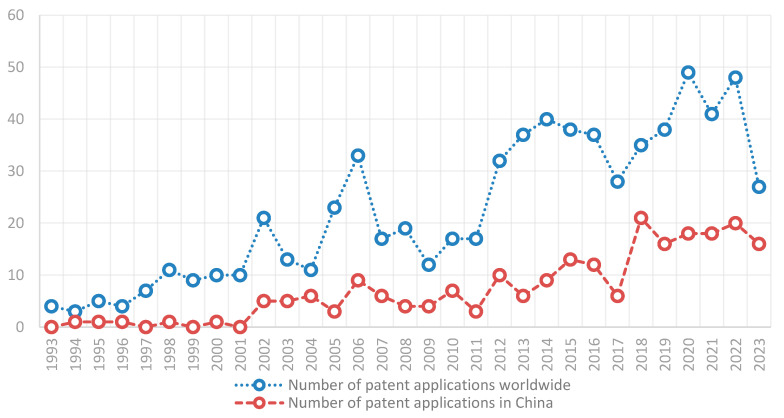
Patent application trends of N2BD technology.

Global patent applications for N2BD started in 1954. However, patent applications were not filed consecutively each year, with only seven patent families filed between 1954 and 1992. Therefore, we chose to analyze the years 1993–2023, during which there were continuous yearly applications, as shown in Figure 4.

From the perspective of global patent application trends, the continuous application of patent delivery began in 1993. From 1993 to 2004, the number of applications was small and grew slowly, generally remaining in the single digits. Since 2005, the number of applications increased significantly, showing an overall upward trend.

From the perspective of Chinese patent applications, patent applications started in 1994, but the patent applications started continuous patent application until 2002; from 2002 to 2017, the number of patent applications increased to about 10 per year. The patent applications increased in 2018, and then remained at about 20 per year.

Considering the time lag between patent filing to disclosure (up to 30 months, including a 12-month priority period and an 18-month disclosure period), the number of patents between 2021 and 2023 is inconsistent with the actual situation, so it does not fully represent the application trend of the three years.

###### Technology Life Cycle

The technology life cycle means that technologies, from being proposed to being eliminated, go through four stages: germination, growth, maturity, and recession [32,33].

**Figure 5:**
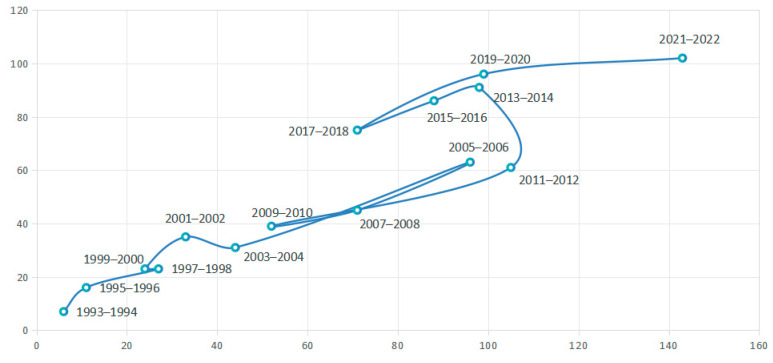
Lift cycle chart of N2BD technology.

The figure below illustrates the life cycle diagram of global N2BD patent technology (Figure 5). It is depicted with two years as nodes, where the number of patent applicants is shown on the horizontal axis and the number of patents on the vertical axis. This diagram highlights the evolution of global N2BD patent technology by tracking the relationship between the changing number of patent applicants and patents annually.

N2BD technology patent applications began in 1954, with continuous filings emerging in 1993. The technology underwent a prolonged germination phase (1954–1996), followed by an initial growth period (1997–2006). Subsequently, a brief downturn occurred (2007–2010). Despite overcoming technological hurdles, the influx of patent applicants led to marginal output growth (2011–2012). Some participants later exited the competition, resulting in a declining number of applicants, while patent applications remained relatively stable, indicating a period of steady growth (2013–2018). From 2019 to 2022, a rapid upward trajectory was observed, with both patents and applicants increasing significantly, hinting at an imminent surge in N2BD technology. However, due to the lag between patent application and disclosure, data from 2021 to 2023 may be distorted, and hence were excluded from the analysis.

#### 3.3.2. Area Layout

##### Source Countries of the Technology

The patented technology output capacity of a country or region is indicated by the number of patents in the country or region where the patent applicant is located, and the higher the percentage of patents, the greater the patented technology output capacity of the country or region in the field of N2BD technology.

**Figure 6:**
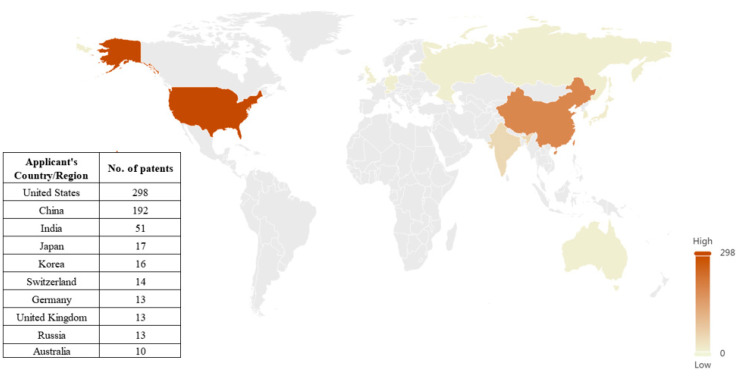
Source countries of N2BD technology.

From the perspective of the distribution of technology source countries (Figure 6), research and development of N2BD patent technology is mainly concentrated in the United States and China. The American patent applications hold the absolute leading position with 298 patents, accounting for 42.3%. The output capacity of N2BD patent technology in China is ranked second with 192 patents, accounting for 27.2%, and in third place is India, with 51 patents, accounting for 7.2%. In addition, Japan, South Korea, Switzerland, Germany, the United Kingdom, Russia, and Australia also have certain patent applications, but the number of patents is fewer than 20, accounting for a relatively small proportion. It can be seen that the United States and China are the main sources of technology in the field of N2BD patents.

##### Target Market Countries

The target market is reflected in the number of patents disclosed by countries, and the higher the percentage of patents, the more market attention the country or region receives in the field of N2BD technology.

**Figure 7:**
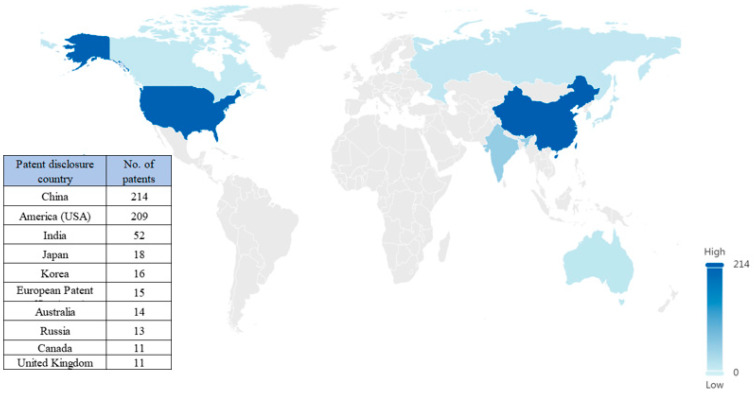
Target markets for N2BD.

From the distribution of target market countries (Figure 7), China and the United States are the two countries with the most market attention to N2BD technology, and the number of patents in the two countries is close to each other, with 214 patent families in China, accounting for 30.4%, and 209 patent families in the United States, accounting for 29.7%. Additionally, there are other target market countries or regions, such as India, Japan, South Korea, Europe, and so forth.

#### 3.3.3. Leading Institutions

From the perspective of patent applicants for N2BD technology (Table 3), the top 10 types of institutions mainly include enterprises, universities, hospitals, and non-profit healthcare organizations. Among these, five are enterprises, three are universities, one is a hospital, and one is a non-profit healthcare organization. The number of patents applied for by each organization in the top 10 is not concentrated, with no single organization having a significantly higher number of patents. This indicates active engagement in research and development within the field, demonstrating a willingness to invest in technological advancement. N2BD technology is shown to be in a phase of gradual development. Regarding the timeline of patent applications, 9 out of the top 10 applicant organizations began their research on N2BD technology after 2001. Among these, three organizations initiated research within the last decade, while six organizations have been actively researching in the past five years.

#### 3.3.4. Technical Composition

**Figure 8:**
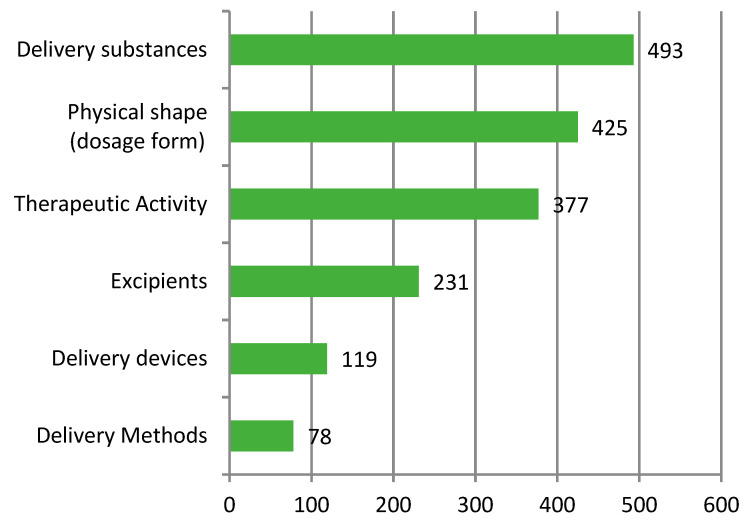
Composition of the patented technologies for nose-to-brain delivery.

After literature research and combining it with the interpretation of patent classification numbers, the composition of patent technologies for N2BD could be categorized into six types: physical shape (mainly manifested as dosage form; A61K9), delivery substances (A61K31, A61K38, A61K36, A61K35, A61K39, and A61K48), excipients (A61K47), therapeutic activity (A61P), and delivery devices (A61M), as well as delivery methods. Delivery methods of N2BD are currently a hot topic of interest and are not reflected in the patent classification numbers for N2BD technologies (Figure 8).

##### Physical Shape

Drugs can be challenging to deliver directly through the nasal route due to solubility issues, stability concerns, and poor bioavailability. Therefore, modifications are often necessary to enhance their physicochemical properties. For instance, drugs are commonly administered nasally as solutions or powders that must dissolve for absorption in the nasal mucosa. While lipophilic drugs can easily cross the nasal mucosa, their water solubility is limited. To address this, they can be administered as more water-soluble precursors, allowing for the preparation of nasal solutions with adequate concentrations.

The absorption of drugs in the nasal cavity can be influenced by changing the physical shape (usually presented as dosage form) of the drug formulation to regulate the retention time of the drug in the nasal cavity and the degree of contact between the drug and the nasal mucosa. Through the classification number of physical shape patents of N2BD technology (Table 4), the common physical shapes of nose-to-brain drug delivery patents (usually presented as dosage forms) include A61K9/12 (aerosol or foam), A61K9/06 (ointment or gel), A61K9/08 (solutions), A61K9/127 (liposomes), A61K9/51 (microspheres or microcapsules), A61K9/107 (emulsions), A61K9/14 (fine granules, e.g., powders), A61K9/72 (for smoking or inhalation), A61K9/16 (lumps, granules, or micro-pearls), A61K9/70 (reticulate, flake, or filamentary base), etc.

##### Delivery Substances

Organized by patent classification numbers of N2BD technologies, the main substances delivered by nose-to-brain pharmaceutical preparations are as follows (Table 5): A61K31 (pharmaceutical preparations containing organic active ingredients), A61K38 (pharmaceutical preparations containing peptides), A61K36 (pharmaceutical preparations containing an undetermined structure derived from algae, mosses, fungi, or plants, or derivatives thereof, e.g., traditional herbal medicines), A61K35 (pharmaceutical preparations containing substances of undetermined composition or reaction products thereof), A61K39 (pharmaceutical preparations containing antigens or antibodies), and A61K48 (pharmaceutical preparations containing the genetic material, where said genetic material is inserted into a cell of a living organism for the treatment of hereditary diseases, and gene therapy).

##### Excipients

The absorption process of nasal drug administration is limited by two factors: the drug’s low membrane permeability and the removal of nasal mucosal cilia [34]. Currently, the most common approach to improving the membrane absorption of the active drug or reducing the clearance of nasal mucosal cilia to increase drug bioavailability and enable the drug to exert maximum therapeutic effects is to search for functional excipients in formulation prescriptions, in addition to the development of suitable delivery devices [35].

Excipients for N2BD techniques are divided into four main categories by patent classification number (Table 6): organic compounds, macromolecular organic or inorganic compounds, inorganic compounds, and modifiers.

In recent years, researchers have focused on the development and application of two types of excipients: bioadhesives and absorption enhancers (see Table 7). For peptide drugs, enzyme inhibitors can protect them from enzymatic degradation, thereby prolonging their duration of action. The use of immune adjuvants has become crucial in enhancing the bioavailability of nasal drugs. This is particularly important due to the outbreak of novel coronaviruses in late 2019, which pose a significant threat to global public health security. As a result, there is now a greater emphasis on the development of nasal vaccines.

##### Delivery Device

To fully utilize the benefits of N2BD and facilitate rapid delivery of active ingredients to the brain or target delivery site, drug delivery devices should aim to deliver drugs to the upper nasal cavity. This area includes the olfactory and upper respiratory regions. Therefore, drug delivery devices that can specifically target the upper nasal cavity are crucial for delivering drugs to the brain via the nose.

The patent classifications for nasal and brain delivery technology mainly use inhalers and sprayers/nebulizers as delivery devices (Table 8). The inhaler technology is divided into an inhalation device inserted into the nose, an aerosol tank, an inhaler interface, a mechanical respiratory trigger (activated through exhalation), and inhalers with a dose or measuring device, with an airflow adjustment device, ultrasonic use, inhalation room, etc. Sprayers/nebulizer technology is divided into air pressure operation applied to the liquid to spray or atomized (or other products), syringe- or piston-type sprayer or atomizer, injector type, use of ultrasonic wave, operation by applying mechanical pressure to the liquid to be sprayed or atomized, and steam pressure operation applied to the liquid to be sprayed or atomized.

The main nasal devices used in N2BD technology patents are shown in Table 9.

##### Delivery Method

The blood–brain barrier (BBB) is a significant obstacle in the pharmacological treatment of CNS diseases. It serves to protect the brain from harmful substances, such as bacteria and viruses, but also restricts the entry of drugs into the brain tissue, limiting their efficacy.

Various invasive techniques have been reported in the literature for trans-BBB drug delivery [4,7,9]. These include the use of ultrasound or chemical means to disrupt the BBB tight junctions, intracranial drug delivery through direct administration of drugs into the ventricles, intracerebral or intrathecal delivery after craniotomy, convection-enhanced drug delivery, and brain implantation using polymers or microchips. The invasive trans-BBB drug delivery strategy has the advantage of directly reaching brain tissue to exert drug effects. However, it is prone to causing structural damage to the BBB, which can result in plasma proteins leaking into the brain and causing neurotoxicity, neuroinflammatory reactions, and other chronic neuropathological changes. Additionally, it may also increase the risk of intracranial infections, brain tissue injuries, and thrombosis.

The literature [2,4,6,13,17] has reported that non-invasive brain-targeted drug delivery refers to the use of the endogenous transport mechanism of the BBB to deliver drugs to the brain tissue, particularly based on cellular carriers, cell-penetrating peptides, specific receptors, and nano-crosslinked drug delivery. This strategy has shown promising applications in the treatment of CNS diseases due to its ease of use, improved safety, and higher BBB delivery efficiency.

**Figure 9:**
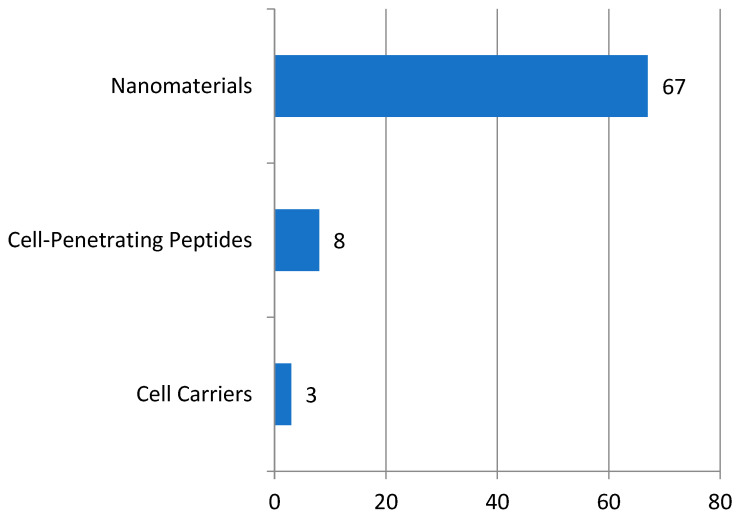
Main delivery modes for N2BD technologies.

Current hotspots of concern in the literature research on N2BD technology include delivery methods that are not reflected in the patent classification numbers. Therefore, we provided an analysis of the delivery methods through manual collation. Based on the above classification, nose-to-brain drug delivery technology primarily involves invasive methods, such as ultrasound and surgery, to penetrate the blood–brain barrier, as well as non-invasive methods, such as cell carriers, cell-penetrating peptides, and nano-cross-material drug delivery. The number of patents for invasive blood–brain barrier (BBB) drug delivery technology for N2BD is limited. This section analyzes non-invasive brain-targeted drug delivery modes of N2BD technology patents, including cell carriers, cell-penetrating peptides, and nano-cross-material drug delivery modes. Figure 9 shows that in the field of major non-invasive brain-targeted drug delivery modes, the most numerous patents are for nanomaterial drug delivery, followed by cell-penetrating peptides, and cellular carrier patents are less numerous.

#### 3.3.5. Technical Hotspot

**Figure 10:**
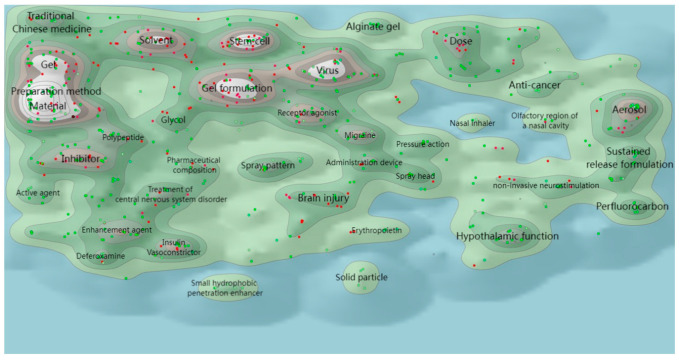
ThemeScape map of patent subjects for N2BD technologies.

In this section, the hotspot analysis of N2BD technology is carried out from the perspective of patent technology theme clustering and hotspot migration. Figure 10 shows the global patent technology theme clustering of N2BD, which is a technology clustering map based on the technology-related patent titles and abstracts of global N2BD, and the technology clustering map using the ThemeScape patent map function in the DI database [36]. Theme clustering groups similar subject records together, forming peaks of varying volume according to the density of the subject literature. The height of the peaks represents the density of the literature records, and the distance between the peaks represents the relationship of the literature records in the region, where the closer the distance, the more similar the content [37]. The red dots on the map represent recent patented technologies disclosed in 2021–2023, and the green dots represent early patented technologies whose disclosure period is before 2020. It can be seen that gel technology, material preparation methods, solvents, and stem cell technology are the focus of attention for N2BD technology.

**Figure 11:**
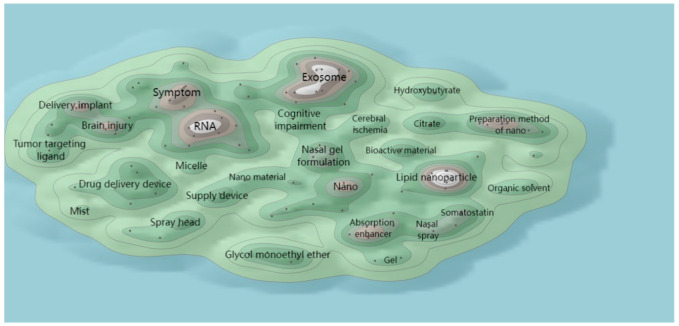
ThemeScape map of N2BD technology patents in the last three years.

The patent technology topics of global N2BD technology in the last three years (2021–2023) were clustered to gain a better understanding of the hotspots of this delivery technology. The research hotspots of N2BD technology were found to be exosome, RNA, and nanotechnology (Figure 11).

**Figure 12:**
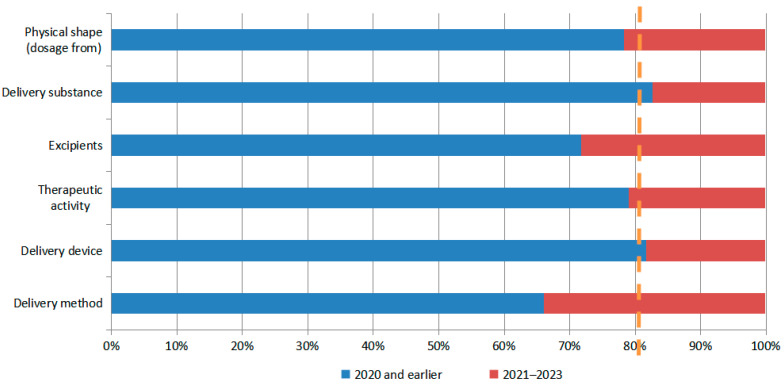
Hotspot migration of nose-to-brain delivery patent technology.

Figure 12 presents patented technologies disclosed between 2021 and 2023 (indicated in red) to represent near-term technologies, and those disclosed before 2020 (indicated in blue) to represent early-stage patented technologies. This categorization allows for a clear representation of patent disclosures in the near-term and early stages, respectively, for N2BD technologies. The figure shows that 79% of the total number of patents were early patents (blue), with a baseline drawn at the 79% position of the horizontal coordinate. Recent technologies exceeded the baseline, indicating the migration of technology hotspots (Figure 12). The patents for N2BD technology show rapid development in the physical shape (dosage from), excipients, and delivery methods. This is particularly true for the delivery method, which is a current focus.

**Figure 13:**
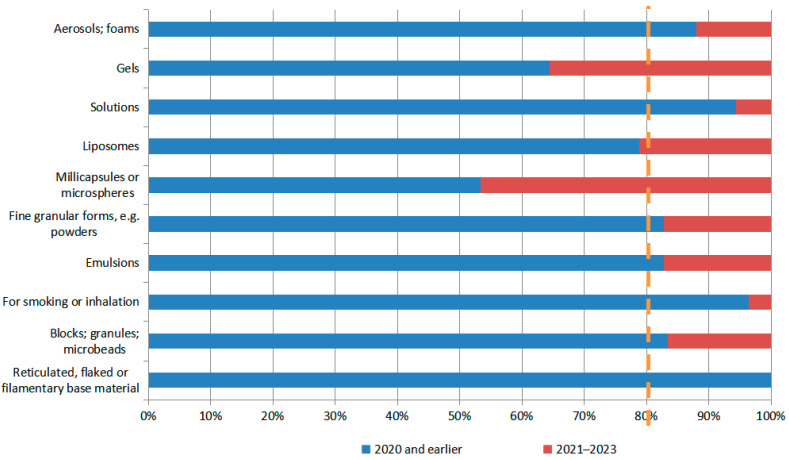
Hotspot migration of physical shape (dosage form) for N2BD.

**Figure 14:**
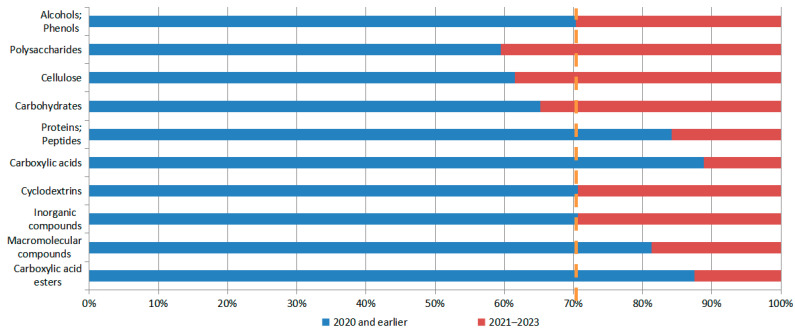
Hotspot migration of excipients for N2BD.

**Figure 15:**
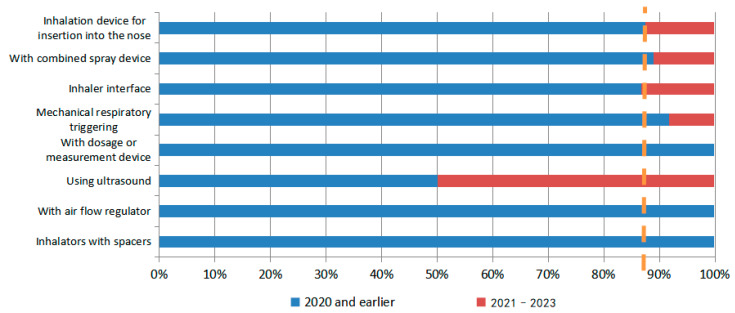
Hotspot migration of inhalers for N2BD.

**Figure 16:**
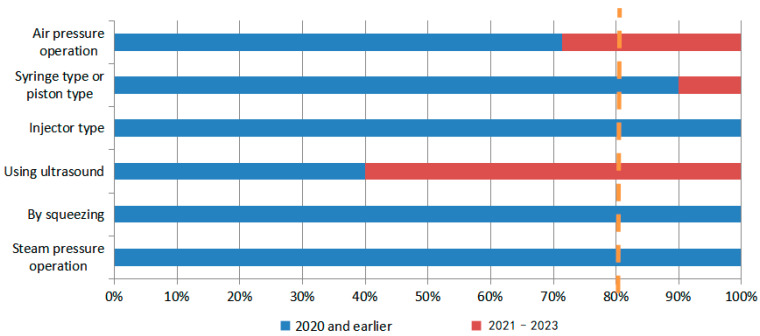
Hotspot migration of nebulizers for N2BD.

Patent hotspot migration analyses of the physical shape (dosage form), excipients, and delivery methods (inhalers and nebulizers) of N2BD technologies were each performed concerning the above methods (Figure 13, Figure 14, Figure 15 and Figure 16). As can be seen from Figure 13, in terms of the physical shape (dosage form) of N2BD technology, the recent patents for gels and microcapsules (microspheres) show a relatively fast pace of development, while the recent patents for liposomes have exceeded but are close to the baseline, which may be of continuing concern.

Figure 14 illustrates the hotspot migration of excipients for N2BD. It shows that patents on polysaccharides, their derivatives (e.g., gum, starch, alginate, dextrin, hyaluronic acid, chitosan, inulin, agar, or pectin), and cellulose and carbohydrates (e.g., sugar alcohols, monosaccharides, nucleic acids, disaccharides, or oligosaccharides) have been granted in recent years. For example, sugar alcohols, monosaccharides, nucleic acids, disaccharides, or oligosaccharides, and their derivatives (e.g., polysorbate, dehydrated sorbitol fatty acid esters, or licorice sweeteners), have developed at a relatively rapid pace in recent times. The recent patents on alcohols, phenols (e.g., glycerol, polyethylene glycol (PEG), poloxamer, and PEG/POE alkyl ethers), cyclodextrins, and inorganic compounds have not exceeded the baseline, but are located near the baseline and could be the subject of sustained attention.

Regarding the migration of hotspots for delivery devices in N2BD patent technology (as shown in Figure 15 and Figure 16), ultrasonic technology has emerged as the leading direction for delivery devices, such as inhalers and nebulizers. Recent patents have shown significant development in this area. The specific patents are Patent Application No. US16229066, which describes an olfactory delivery apparatus, system, and methods for delivering pharmaceutical preparations by converting liquids into nano-emulsions through ultrasonic cavitation. Patent Application No. CN202210063935.9 describes a delivery system for administering drugs to the brain through the nasal olfactory region. The nebulizer uses high-intensity ultrasonic waves of 1–10 MHz to dispense drugs in the drug reservoir, forming particles or droplets with a particle size of 200 nm or less. Patent Application No. CN202310362512.1 describes a comprehensive intervention system for brain diseases that combines hydrogen or oxygen absorption and drug delivery through the nasal–brain route. The system includes an ultrasonic nebulizer for drug delivery.

In summary, the above migration analysis of hotspots of N2BD patents indicates that physical shapes (dosage form), excipients, and delivery methods have undergone a relatively rapid level of development in recent times. The research hotspots of physical shape (dosage form) of N2BD technology in the last three years are gels and microcapsules (microspheres). The research hotspots of excipients for N2BD technology in the last three years are polysaccharides and their derivatives (e.g., chitosan, hyaluronic acid, inulin, and pectin), alcohols, phenols (e.g., polyethylene glycol (PEG) and zonisamide), and cellulose and its derivatives. The research hotspots for delivery modes are mainly focused on nanotechnology, including nanoparticles, nano-emulsions, lipid nanoparticles, and nanogels.

#### 3.3.6. Topic Evolutionary Analysis

##### Topic Evolutionary Analysis Based on the LDA Model

The theme evolution relationship model proposed by Nagehan İlhan [38] classifies the evolution relationship into six types: newborn, survive, dissolve, merge, shrink, and split. The above temporal theme evolution relationship was extracted by calculating the cosine similarity between the themes of the neighboring time windows and setting a threshold. The specific determination conditions are shown in Table 10.

The objects of study in the evolution of patent technology topics in this study were the titles and abstracts of patents. Patent titles and abstracts, as a high-level summary of patent literature, cover a wealth of technological innovation information [23]. The patent data were categorized into 1990–1994, 1995–1999, 2000–2004, 2005–2009, 2010–2014, 2015–2019, and 2020–2023, covering seven time windows. The topic evolution model construction was realized based on Python (version number 3.9.0), and the text preprocessing was carried out using “Jieba” Chinese text segmentation [39]. Via the perplexity of the patent data for each time window, the optimal number of topics for each time window was determined. LDA topic clustering analysis was carried out on the patent data within each time window to generate the topics for each time window, as presented in Table 11.

**Figure 17:**
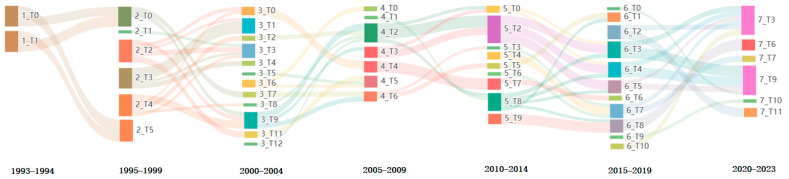
Thematic evolutionary pathway map of N2BD.

The cosine similarity was employed to correlate the topics in the neighboring time windows, with a cosine similarity threshold of 0.3. If the cosine similarity between two topics is ≥0.3, there is an evolutionary relationship between the topics. The theme evolution relationship was plotted as a Sankey diagram using the PyCharts library, with the results shown in Figure 2. The connecting line in the graph represents the direction of technological innovation flow, and the thickness of the connecting line represents the degree of correlation between the themes. The closer the degree of correlation is, the thicker the connecting line is. Thematic evolutionary relationships were plotted as Sankey diagrams using the PyChart charting class library, and the results are presented in Figure 17.

As illustrated in Figure 17, there were six evolutionary relationships among the N2BD technology themes: newborn, survive, dissolve, merge, shrink, and split. There were also large differences in the strength of the evolutionary capacity of the research themes over time.

From 1990 to 1994, only seven patents were filed, mainly for compounds. The subject matter revolved around improving hypothalamic function through nasal administration, and the main keywords included: compounds, hypothalamus, steroids, and estrogenic steroids. From 1995 to 1999, there were four new themes, one dissolved theme, and one surviving theme. The four newborn themes were 2_T1 (Calcitonin and Polymer), 2_T2 (Intranasal Administration of Vitamin B12), 2_T3 (CNS and Migraine Headache), and 2_T4 (CNS and Vitamins). The theme of dissolution was 2_T5 (Nasal Administration of Progesterone to the Hypothalamus). In contrast, the surviving theme was 2_T0 (Intranasal Administration of Steroids). From 2000 to 2004, there were more split themes, and the top three themes in terms of thematic intensity were 3_T9 (CNS and Dopamine Agonists), 3_T1 (Migraine and Alzheimer’s disease), and 3_T3 (Transnasal Therapeutic Agents for Migraine Headaches). Emerging topics are 3_T5 (Herbal Medicine Transnasal Treatment for Ischemic Brain Disease), 3_T6 (Nasal Spray, Hyperosmolar Sugar Composition, and Plant Essential Oil), and 3_T12 (DELTA-9-Tetrahydrocannabinol (THC) Delivery).

During the period 2005–2009, the top three themes in terms of theme strength were 4_T2 (CNS, Peptides, and Obesity), 4_T4 (Vitamin B12 and Blood–Cerebrospinal Fluid Barrier), and 4_T5 (Nasal Spray, Herbal Rhinitis, and Clenbuterol). During this period, a newborn topic was 4_T1 (Sialic Acid and Recombinant Human Erythropoietin), typically patented as ‘Rh-epo nasal formulations with low sialic acid concentration for the treatment of diseases of the central nervous system’ (CN201510674598.7). The theme of dissolution was 4_T0 (ACE Inhibitors and Blood–Brain Barrier), typically patented as ‘Compositions and methods using acetylcholinesterase (ACE) inhibitors to treat central nervous system (CNS) disorders in mammals’ (US11112950). During this period, a new theme emerged, labeled as 4_T0 (ACE Inhibitors and Blood–Brain Barrier), with a typical patent being the patent for ‘Compositions and methods using acetylcholinesterase (ACE) inhibitors to treat central nervous system (CNS) disorders in mammals’ (Patent Number: US11112950). During the period 2010–2014, the three newborn themes were 5_T4 (Sem Grafts, Depression, and Parkinson’s Disease), 5_T6 (Antibiotics and Brain Injury), and 5_T9 (Polymer and Nanoparticles). The top three themes in terms of theme strength were 5_T2 (CNS, Liposomes, and Nasal Powder), 5_T8 (CNS, NSAIDs, and Alzheimer’s Disease), and 5_T7 (Vitamin B12 and CSF). During the period 2015–2019, there were four newborn themes, one dissolved theme, and one surviving theme. The four newborn themes were: 6_T0 (Disposable Transnasal Brain-Targeted Drug Delivery Device), 6_T2 (Chitosan, Stem Cells, and Alzheimer’s Disease), 6_T9 (Transnasal Microemulsions), and 6_T10 (Stem Cells). During the period 2020–2023, the top three themes in terms of theme strength were 7_T3 (Stem Cell, CNS, and mRNA), 7_T9 (CNS and Olfactory Region Delivery), and 7_T6 (Polymers, Liposomes, and Aromatic Dialdehydes), with the majority being in the merged themes. It is worth noting that two new themes were emerging during this period, one related to new coronaviruses and the other to exosomes. It can be seen that with the COVID-19 pandemic, nasal administration of drugs for the treatment of novel coronaviruses has become one of the hotspots.

A comprehensive analysis of the entire period from 1990 to 2023 revealed five principal evolutionary paths related to nose-to-brain delivery technologies. The first route is 1_T0→2_T0→3_T7→4_T6→5_T3→6_T7→7_T3. The second route is 1_T0→2_T0→3_T9→4_T2→5_T2→6_T3→7_T3. The third is 1_T0→2_T0→3_T7→4_T6→5_T3→6_T7→7_T7. The fourth is represented by the sequence 1_T0→2_T0→3_T9→4_t2→5_T8→6_T4→7_T3. The fifth is 1_T0→2_T0→3_T9→4_t2→5_T8→6_T8→7_T9. Of these, Route 1 reflects a trend in the indications for the N2BD technology, which has evolved from the treatment of rhinitis through the regulating steroid hormones in the hypothalamus to the treatment of migraine, Parkinson’s disease, Alzheimer’s disease, and depression. Route 2 reflects evolutionary trends in delivery modalities, which have progressed from liposomes to nanoparticles to exosomal drug delivery. Route 3 reflects the evolutionary trend of delivery devices, which have evolved from multi-dose delivery to single-dose delivery to precise olfactory zone delivery modalities. Route 4 reflects the evolution of delivery media, which have evolved from small molecules (e.g., vitamin B12) to large molecules (e.g., vaccines) to stem cells. Route 5 reflects the changes in delivery excipients, which have evolved from simple cellulose and sugar alcohols to polyethylene glycol, poloxamer, and chitosan.

##### Technology Evolution Path

**Figure 18:**
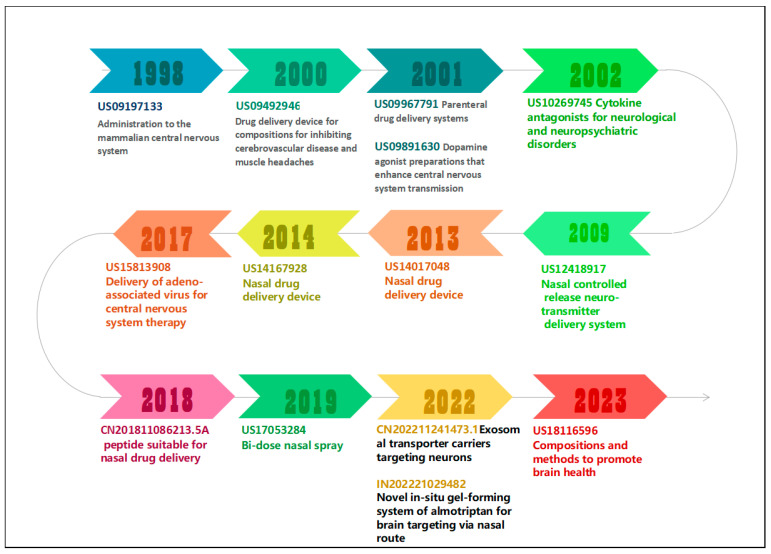
Evolution path of N2BD technologies.

Figure 18 illustrates the technology evolution path of N2BD technology from 1998 to 2023. This paper characterized the development of N2BD technology through key patents and operational indicators, such as citation frequency and patent value. The patent value was calculated using IncoPat’s self-developed model, which integrates common and important technical indicators in the patent analysis industry. The patent value model includes the most significant technical indicators in the patent analysis industry, such as technological stability, technological advancement, and scope of protection. By setting the weights of the indicators, the order of calculation, and other parameters, the model assigns a score of 1–10 to each patent. The higher the score, the higher the patent value. The 15 patents with high citation frequency and patent value were selected as the core patents in this field. The development of N2BD technology was learned from these patents.

The 15 core patents ranged from 1998 to 2023. Previous core patents were primarily from foreign entities, including BHL Patent Holdings LLC, TACT IP LLC, MetP Pharma AG, Temple University, Impel, OptiNose, and the University of Minnesota. In contrast, recent core patents appear to have been filed by domestic universities, such as Peking University and Xuzhou Medical University.

In 1998, Intrabrain International Inc. filed a patent application (Application No. US09197133 [40], cited 130 times) for drug delivery to the central nervous system of mammals. The application discloses a device, method, and pharmaceutical composition for combining electrode delivery or ultrasonic infiltration therapies with chemical permeation enhancers for transnasal delivery of a drug to the CNS. The method utilizes a physical enhancement device and a chemical enhancement method to deliver a drug to the nervous system from a remote site that corresponds to a distal end of a neural pathway.

In 2000, BHL Patent Holdings LLC, USA, disclosed a drug delivery device (Application No. US09492946 [41], cited 369 times) for inhibiting cerebrovascular disease and muscle headaches. The device has an elongated, curved shape and is inserted into the patient’s nostrils by manual pressure.

In 2001, Nastech Inc. in the United States disclosed dopamine agonist formulations to enhance CNS delivery (Application No. US09891630 [42], cited 148 times). The formulations include one or more mucosal delivery enhancers selected from (a) a substance that inhibits aggregation; (b) an agent that modifies charge; (c) a substance that controls pH; (d) an inhibitor of degradative enzymes; (e) substances that break down or remove mucus; (f) substances that inhibit cilia; (g) substances that enhance membrane permeability; (h) substances that modulate epithelial junction physiology; (i) substances that dilate blood vessels; (j) substances that enhance selective transport, and (k) vehicles that stabilize delivery. These methods and formulations provide significantly enhanced uptake of dopamine receptor agonists in or across the nasal mucosal barrier to the target site of action, e.g., in the central nervous system.

In 2001, Naito Albert T of the United States disclosed a parenteral drug delivery system (Application No. US09967791 [43], cited 158 times). The patent facilitates the crossing of the blood–brain barrier by co-administering a nutritive, diagnostic, or therapeutic agent with a hyperosmolar sugar composition that permits administration of the agent via absorption into the body through the nose or lungs.

In 2002, TACT IP LLC, USA, developed a cytokine antagonist for the treatment of neurological and neuropsychiatric disorders through intranasal administration (Application No. US10269745 [44], cited 128 times). The recommended dosage is 1–50 mg per dose, with administration intervals as short as 4 h. The target indications for this treatment include depression, schizophrenia, anorexia nervosa, and chronic fatigue syndrome.

In 2009, MetP Pharma AG (formerly Mattern Pharmaceuticals) of Switzerland disclosed a nasal controlled-release delivery system for neurotransmitters (application number US12418917 [45], cited 83 times). The invention’s special lipophilic or partly lipophilic system results in high bioavailability of the active ingredient in plasma and the brain due to sustained serum levels and/or direct or partly direct transportation from the nose to the brain.

In 2012, Impel NeuroPharma Inc. (USA) disclosed a drug delivery device titled “nasal drug delivery device”, which is a pressurized olfactory device for direct nose-to-brain drug delivery. The pressurized olfactory device (POD™), also called a precision olfactory device, is an aerosol nasal spray with a narrow spray plume with circumferential velocity. The POD system utilizes the rich vasculature found in the olfactory region of the upper nasal space to provide consistent and predictable drug delivery and improve bioavailability. It is a handheld, manually actuated, gas-propelled administration device designed to deliver active drugs, specifically to the upper nasal space [46].

In 2014, OptiNose disclosed a delivery device and method for providing substance delivery to the central nervous system (CNS) of a subject (Application No. US14167928 [47], cited 39 times). The device achieves this by creating positive pressure in the oral cavity, for example, through oral exhalation. The efficacy improvement achieved by this invention compared to existing nasal spray drug delivery systems can be attributed to the fact that it delivers mainly to the anterior third of the nasal airway, which is the nasal region in front of the nasal valve.

In 2017, the University of Minnesota disclosed the use of adeno-associated virus for therapeutic delivery to the central nervous system (Application No. US15813908 [48], cited 18 times). The adeno-associated viral vectors (AAV) used in the methods of the invention can be employed to deliver genes to the CNS. One embodiment of the invention involves delivering therapeutic proteins to the CNS through an adeno-associated virus (AAV) via intranasal administration. This method aims to prevent, inhibit, or treat neurocognitive dysfunction or neurological disorders.

In 2018, the Shenzhen Graduate School of Peking University disclosed the use of a peptide in the preparation of drugs for intravenous, intraperitoneal, or nasal administration (Application No. CN201811086213) [49]. The study found that a fusion peptide capable of passing the blood–brain barrier can be obtained by connecting the C-terminal end of ziconotide to the N-terminal end of the cell-membrane-penetrating peptide. This peptide is suitable for intravenous, intraperitoneal, or nasal administration and has a good analgesic effect and long duration of efficacy in vivo. It can be used on a large scale in the clinic. The patent value degree is 10, and it has been cited 9 times.

In 2019, Akroswiss in Switzerland disclosed an application for a dual-dose nasal spray (Application No. US17053284 [50], cited once, patent value degree = 10). The cited patent describes a nasal spray that administers an equal, defined volume of an active agent solution or liquid intranasally to a patient. The nasal spray can be used in any body position, independent of the spatial orientation of the spray. The patent emphasizes the importance of equal dosing and ease of administration for patients.

In 2023, Biosciences LLC of the USA disclosed a composition and method for promoting brain health (Application No. US18116596 [51], cited once, patent value degree = 10). The active ingredient, D-beta hydroxybutyrate, is encapsulated in biodegradable polymer particles and administered nasally for controlled release. This method bypasses the blood–brain barrier and is used for neuroinflammation. The polymer is made up of a mucosal adhesion polymer and a thermoreversible polymer. Mucosal adhesion polymers such as chitosan, alginate, and cellulose derivatives, as well as thermoreversible polymers such as gelatin, carrageenan, hydroxypropyl methylcellulose (HPMC), and xyloglucan, are commonly used.

**Figure 19:**
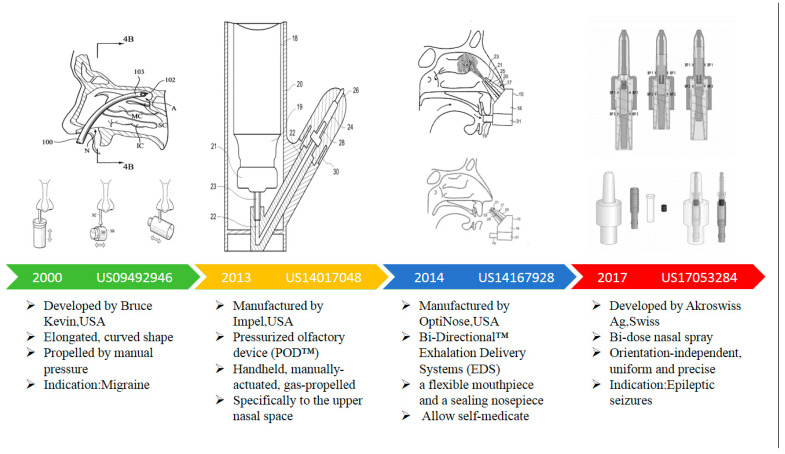
Patent attachments for the main for nose-to-brain devices [48,49,50,51].

Among them, Patent Application Numbers US09492946, US14017048, US14167928, and US17053284 are all devices for nose-to-brain delivery. The accompanying drawings of the devices are shown in Figure 19.

US09492946 of 2000 is a drug delivery device with an elongated, curved shape that is propelled by manual pressure. In 2012, Impel NeuroPharma Inc. disclosed a device that uses pressurized gas as a propellant to deliver drugs to the olfactory region of the nasal cavity (Patent No. US140,170,048), known as the pressurized olfactory device (POD™), also called a precision olfactory device. POD™ comprises a canister capable of containing a propellant, a diffuser in communication with the canister, a compound chamber in communication with the diffuser, and a nozzle in communication with the compound chamber. The POD™ device is generally evaluated for desired actuation volume, spray rate, particle size and size distribution, and nasal cavity deposition efficiency. In 2014, OptiNose Inc. developed a technology platform for bi-directional expiratory delivery systems (US14167928) and already has two marketed products, Xsail^®^ and Xhance^®^. Xsail^®^ uses a disposable, single-use nosepiece that is attached by the patient to the body of a delivery device, already fitted with a mouthpiece and a piercing mechanism. The design of the Xsail^®^ Breath-Powered Delivery Device harnesses the patient’s own breath to seal off the nose from the throat and deliver a low dose of a trusted medication to the richly vascular passages deep in the nose [52]. Both POD™ (US14017048) and Xsail^®^ (US14167928) are devices designed to deliver drugs to specific regions of the nasal passages. However, they use different technical solutions. The former delivers the drug via HFA propellant from a pressurized tank, while the latter uses oral inhalation. Each of the dual-dose sprays of US17053284, 2019, contain an equal and defined volume of active agent solution or liquid. The nasal spray allows for administration in any body position of the patient.

In nasal sprays, common drug delivery devices generally deliver drugs to the vestibular and lower respiratory regions. However, one of the difficulties in N2BD is how to deliver the drug to the upper nasal cavity and even to the olfactory region in a precise and quantitative way [14,15]. Scientists all over the world have conducted a lot of research on optimizing the drug delivery devices, and a large number of patents have been generated, some of which have helped to bring products to market for the benefit of patients [8,9,53,54]. Taking Figure 19 as an example, N2BD devices have undergone significant evolution, progressing from elongated curved devices inserted into the nostrils in the early stages to region-specific precision delivery devices, and to precise bi-dose and single-dose drug delivery devices. 

The nose-to-brain delivery route has also been found to have limitations, such as a relatively small delivery volume, limited surface area of the olfactory epithelium, short residence time for drug absorption, and the presence of nasal mucosal clearance [4,18,34]. Moreover, both the spray device and the nature of the contents have a critical influence on the spray pattern, spray morphology, and particle size. To further improve upper nasal and olfactory region deposition and prolong the drug residence time in the olfactory region, scientists have further used drug delivery devices in conjunction with new formulation and delivery technologies to further meet the needs of nose-to-brain delivery. For example, adding mucosal adsorbents and permeation enhancers to prescriptions, applying nanoparticle formulation technology to prepare drug solutions, etc. [13,55,56], will greatly improve the nose-to-brain pathway delivery performance through the use of nanoparticle formulations in conjunction with drug delivery devices targeting the olfactory region.

### 3.4. Summary of Patent Analysis

N2BD technology has undergone a prolonged period of development. After a brief decline, it entered a stable growth phase. Since 2019, there has been a rapid increase in the number of patents and patent applicants, indicating a trend toward rapid growth in this field. Research and development of patent technology for N2BD are mainly concentrated in the United States and China. These two countries also receive the most market attention for this technology. Patent applicants for N2BD technology are dispersed, with no single organization having a dominant number of patent applications. This suggests that R&D in this field is active and that organizations are investing resources in technology development. It also indicates that N2BD technology is still in the process of gradual development.

From a patent classification number and literature research perspective, the technology composition of N2BD can be broadly divided into six categories: physical shape (dosage form), delivery substance, excipients, therapeutic activity, delivery device, and delivery method. In recent years, there has been rapid development in physical shape (dosage form), excipients, and delivery methods. The research hotspots for physical shapes (dosage forms) are gels, microcapsules, or microspheres. As for excipients, polysaccharides and their derivatives (such as gum, starch, alginate, dextrin, hyaluronic acid, chitosan, inulin, agar, or pectin), alcohols, phenols (such as glycerol, polyethylene glycol (PEG), porosam, and PEG/POE alkyl ether), and cellulose and its derivatives are the main focus. Carbohydrates are also being studied. These delivery modes can be used to transport various compounds, including sugar alcohols, amino-saccharides, ribonucleic acid, mono-, di-, or oligo-saccharides, their derivatives (such as polysorbates, sorbitan fatty acid esters, or licorice sweeteners), cyclodextrins, and inorganic compounds. The delivery modes that are currently being researched mainly focus on nanotechnology, such as nanoparticles, nano-emulsions, lipid nanoparticles, and nanogels. The clustering of patent technology topics revealed that gel technology, material preparation methods, solvents, and stem cell technology are the primary areas of focus for N2BD technology. Exosomes, RNA, and nanotechnology are the current research hotspots for this field.

During the development of nose-to-brain drug delivery technology, various methods have been employed. These include early physical enhancements, such as electrical delivery or ultrasound, chemical enhancement, and the development of nasal sprays. More recent methods include the use of viral vectors, cell-penetrating peptides, exosomes, and nano-in-situ gels. It is important to note that these delivery modes are still being researched and developed. The development of drug delivery devices has come a long way since the early years of placing them in the nostrils using slender, curved devices. Nowadays, there are specific devices designed for precise delivery to certain regions. Multiple types of devices, including multi-dose, double-dose, and single-dose, co-exist. Advancements in technology, materials, and devices have facilitated the progress of N2BD technology.

## 4. Conclusions and Future Outlook

The questionnaire survey results indicated that 165 participants from 28 provinces and 161 different units took part in the survey. The development of nasal formulations in China is still in its early stages. Most interviewees believed that nasal drug delivery has specific clinical needs and market potential. However, the development of domestic nasal formulations faces challenges in terms of device development, sourcing raw materials and auxiliary materials, and ensuring quality control.

Analysis of patents on N2BD technology showed that research on excipients and dosage forms will impact the development of nasal and intracerebral drug delivery. The development and research of transnasal nano-delivery technology can provide a new strategy for the transnasal treatment of central diseases. The nano-delivery system can further cross the barrier of transnasal delivery, giving full play to the advantages of nano-targeting technology to improve the efficiency of drug delivery through nose-to-brain targeting. This optimization of efficacy can reduce adverse reactions. In the future, further exploration is needed on the development of new materials, formulation technology, and transnasal drug delivery devices. It is believed that with technological innovation and cross-disciplinary development, more new technologies, materials, and devices will be applied to N2BD. This will address the existing technological shortcomings, build on the advantages of the technology, and enhance the level of industrialization.

## Figures and Tables

**Table 1:** The top 10 varieties in terms of the number of global marketing licenses.

Rank	Generic Name	ATC Classification
1	Mometasone	Corticoid
2	Xylometazoline	Adreneptor agonists
3	Fentanyl	Phenyloperidol derivatives
4	Fluticasone	Corticoid
5	Azelastine	Non-corticoid antiallergic drugs
6	Dexofenol + Xylometazoline (Combinations)	Adrenreceptor agonist (without corticosteroids)
7	Sumatriptan	Selective serotonin receptor agonists
8	Budesonide	Corticoid
9	Zelastine + Fluticasone (Combinations)	Corticosteroids + non-corticosteroids
10	Desmopressin	Pressin and its analogs

**Table 2:** FDA-approved nose-to-brain formulations.

Active Ingredient	Brand Name	Therapy for	Approval Time	Applicant Holder	Device
Nicotine	Nicotrol^®^	Smoking cessation	1996	Pfizer Inc. (New York, NY, USA)	Reusable nasal spray device
Sumatriptan	Imitrex^®^	Migraine	1997	GlaxoSmithKline (London, UK)	Disposable pre-filled nasal spray device
Dihydroergotamine mesylate	Migranal^®^	Migraine	1997	Bausch Health US LLC (West Laval, QC, Canada)	Nasal spray device
Zolmitriptan	Zomig^®^	Migraine	2003	Amneal pharmaceuticals LLC (Bridgewater, NJ, USA)	Disposable pre-filled nasal spray device
Naloxone hydrochloride	Narcan^®^	Opioid overdose	2015	Emergent Operations Ireland Ltd. (Dublin, Ireland)	Disposable pre-filled nasal spray device
Sumatriptan	OnzetraXsail^®^	Migraine	2016	Currax Pharmaceuticals LLC (Brentwood, UK)	Xsail® system
Sumatriptan	Tosymra^®^	Migraine	2019	Upsher Smith Laboratories LLC (Maple Grove, MN, USA)	Disposable pre-filled nasal spray device
Midazolam	Nayzilam^®^	Epilepsy	2019	Ucb Inc. (Brussels, Belgium)	Disposable pre-filled nasal spray device
Esketamine hydrochloride	Spravato^®^	Depression	2019	Janssen pharmaceuticals Inc. (Raritan, NJ, USA)	Disposable pre-filled nasal spray device
Diazepam	Valtoco^®^	Epilepsy	2020	Neurelis Inc. (San Diego, CA, USA)	Disposable pre-filled nasal spray device
Dihydroergotamine mesylate	Trudhesa^®^	Migraine	2021	Impel pharmaceuticals Inc. (Seattle, WA, USA)	POD® system
Naloxone hydrochloride	Kloxxado^®^	Opioid overdose	2021	Hikma pharmaceuticals usa Inc. (Richmond, VA, USA)	Disposable pre-filled nasal spray device
Varenicline	Tyrvaya^®^	Dry Eye Disease	2021	Oyster Point Pharma Inc. (Princeton, NJ, USA)	Reusable nasal spray device
Zavegepant	Zavzpret^®^	Migraine	2023	Pfizer Inc. (New York, NY, USA)	Disposable pre-filled nasal spray device
Naloxone hydrochloride	Naloxone Hydrochloride	Opioid overdose	2023	Amphastar pharms Inc. (Rancho Cucamonga, CA, USA)	Disposable pre-filled nasal spray device

**Table 3:** Patent applicant for N2BD technology.

Rank	Applicant	No.	Application Renewal Period
1	HealthPartners (Bloomington, MN, USA)	31	2005–2020
2	Nastech Pharmaceutical Company Inc. (New York, NY, USA)	16	2001–2014
3	Pherin Pharmaceuticals Inc. (Mountain View, CA, USA)	15	1993–2001
4	Columbia University in the City of New York (New York, NY, USA)	13	2005–2021
5	OptiNose (Yardley, PA, USA)	13	2006–2019
6	Par Pharmaceutical Inc. (New York, NY, USA)	11	2004–2016
7	Massachusetts Eye&Ear Infirmary (Boston, MA, USA)	8	2012–2022
8	University of Minnesota (Minneapolis, MN, USA)	8	2012–2017
9	Impel Neuropharma Inc. (Seattle, WA, USA)	6	2012–2019
10	Fudan University (Shanghai, China)	6	2002–2020

**Table 4:** Classification of physical shape in N2BD technologies.

Classification Number	Interpretation	Number of Patents
A61K9/12	Aerosol; Foam	58
A61K9/06	Ointments(gel)	45
A61K9/08	Solutions	35
A61K9/127	Liposome	33
A61K9/51	Microspheres, microcapsules	30
A61K9/107	Emulsions	29
A61K9/14	Fine granules, e.g., powders	29
A61K9/72	For smoking or inhalation	28
A61K9/16	Lumps;granulates; microbeadlets, e.g., microparticles	18
A61K9/70	Reticulate, sheet or filament bases	12

**Table 5:** Classification of delivery substances in N2BD technologies.

Classification Number	Interpretation	Number of Patents
A61K31	Pharmaceutical preparations containing organic active ingredients, e.g., amino acids, aryl alkylamines and morphinan derivatives	339
A61K38	Pharmaceutical preparations containing peptides, e.g., Insulin, cellulase, growth factors	134
A61K36	Pharmaceutical preparations containing undetermined structures from algae, mosses, fungi or plants or derivatives thereof, e.g., traditional herbal medicines	61
A61K35	Pharmaceutical preparations containing substances of undetermined composition or reaction products, e.g., hematopoietic stem cells; mesenchymal stem cells; neural stem cells	58
A61K39	Pharmaceutical preparations containing antigens or antibodies, e.g., protease inhibitors, hormones and viral antigens	42
A61K48	Pharmaceutical preparations containing genetic material, said genetic material being inserted into cells of a living organism for the treatment of hereditary diseases; gene therapy	25

**Table 6:** Classification of patent excipients of nose-to-brain delivery technology.

Classification of Excipients	Classification Number	Interpretation	Number of Patents
Organic compounds	A61K47/10	Alcohols; phenols; their salts, e.g., glycerol; polyethylene glycol [PEG]; poloxamer; PEG/POE alkyl ethers	54
	A61K47/26	Carbohydrates, e.g., sugar alcohols, amino sugars, nucleic acids, monosaccharides, disaccharides or oligosaccharides; their derivatives, e.g., polysorbates, sorbitan fatty acid esters or liquorice sweeteners	23
	A61K47/12	Carboxylic acids; their salts or anhydrides	18
	A61K47/14	Carboxylic acid esters, e.g., fatty acid monoglycerides, medium chain triglycerides, parabens or PEG fatty acid esters	16
	A61K47/24	Containing atoms other than carbon, hydrogen, oxygen, halogens, nitrogen or sulphur, e.g., cyclomethicone or phospholipids	16
	A61K47/186	Quaternary ammonium compounds, e.g., benzalkonium chloride or cetrimide	9
	A61K47/20	Containing sulphur, e.g., dimethyl sulphoxide [DMSO], docusate, sodium dodecyl sulphate or sulphamic acid	9
	A61K47/22	Heterocyclic compounds, e.g., ascorbic acid, tocopherol or pyrrolidone	8
	A61K47/28	Steroidal compounds, e.g., cholesterol, bile acids or glycyrrhizic acid	8
	A61K47/183	Amino acids, e.g., glycine, EDTA or aspartame	7
Macromolecular organic or inorganic compounds	A61K47/36	Polysaccharides; their derivatives, such as gum, starch, alginate, dextrin, hyaluronic acid, chitosan, inulin, agar or pectin	34
	A61K47/38	Cellulose; its derivatives	26
	A61K47/42	Proteins; peptides; their degradation products; their derivatives, e.g., albumin, gelatine or zeinolysin	19
	A61K47/40	Cyclodextrins; their derivatives	17
	A61K47/34	Macromolecular compounds not obtained by reactions involving only carbon-carbon unsaturated bonds, such as polyesters, polyamino acids, polysiloxanes, polyphosphonitrile, copolymers of polyalkylene glycols or poloxamers	16
	A61K47/32	Macromolecular compounds obtained by reactions involving only carbon-carbon unsaturated bonds, such as carbomer {poly(meth)acrylate or polyvinylpyrrolidone}	11
Inorganic compounds	A61K47/02	Inorganic compounds	17
Modifiers	A61K47/64	Drug-peptide, drug-protein or drug-polyamino acid affixes, i.e., the modifier is a peptide, protein or polyamino acid covalently bound or complexed with a therapeutically active agent.	15
	A61K47/60	Organic macromolecular compounds are polyalkylene oxide oligomers, polymers or dendritic polymers, e.g., PEG, PPG, PEO or polyglycerol	8
	A61K47/6811	Protein or peptide, e.g., transferrin or bleomycin	7

**Table 7:** Excipients for nasal preparations (by function).

Category	Mechanism of Action	Examples
Absorption enhancers	Increase membrane fluidity and open tight junctions in the intercellular space.	Nonionic surfactants (PEG400, PEG3500, polysorbate 20, polysorbate 80, etc.), cyclodextrins and their derivatives, bile salts (sodium deoxycholate, sodium glycocholate, and sodium taurate), modified vitamin E, polyethylene glycol lithium dodecahydroxystearate, and alkyl glycosides.
Bioadhesives	Slow drug clearance from nasal cilia by increasing the drug residence time through increased viscosity.	Chitosan, hyaluronic acid, and Gellan gum.
Enzyme inhibitors	Protect drugs from enzymatic degradation.	Camostat mesylate, cyclosporine A, and rifampicin.
Immune adjuvants	Enhance the immune response by promoting antigen uptake.	Toxoids, cytokines, and Toll-like receptor agonists.

**Table 8:** N2BD technology patents’ main delivery devices.

Main Delivery Device	Classification Number (IPC/CPC)	Interpretation	No.
Inhalers	A61M15/08	Inhalation devices inserted into the nose	56
	A61M15/009	Use of medication packages with incorporated spray devices, e.g., aerosol cans	18
	A61M15/0021	Inhaler interface	15
	A61M15/0098	Mechanical respiratory triggering—activated by exhalation	12
	A61M15/0065	Inhalers with dosage or measurement devices	9
	A61M15/002	With an airflow regulator	4
	A61M15/0085	Use of ultrasound	4
	A61M15/0086	Inhalation chamber	3
Sprayers or nebulizers specially adapted for therapeutic purposes	A61M11/02	Operated by air pressure applied to the liquid (or other product) to be sprayed or nebulized	14
	A61M11/007	Syringe-type or piston-type nebulizers or atomizers	10
	A61M11/06	Injector type	8
	A61M11/005	Using ultrasound	5
	A61M11/008	Operated by mechanical pressure applied to the liquid to be sprayed or atomized—by squeezing, e.g., using flexible bottles	4
	A61M11/04	Operated by vapor pressure applied to the liquid to be sprayed or atomized	3

**Table 9:** Main nasal devices used in N2BD technology patents.

Nasal Device	Available Dosage Form	Physical Shape	Manufacturer	Main Patent Application No.
Aptar	Nasal drops, nasal spray	Liquid (solution) and powder	Aptar, Crystal Lake, IL, USA	US17625439, US16469703, US17630366, etc.
ViaNase (electronic atomiser)	Nasal spray	Liquid (solution)	Kurve Technology, Lynnwood, WA, USA	/
Precision olfactory delivery (semi-disposable unit dose delivery)	Nasal spray	Liquid (solution) and powder	Impel Pharmaceuticals, Seattle, WA, USA	US14017048
SipNose (pressurized delivery)	Nasal spray	Liquid (solution)	Sipnose, Yokne’am Illit, Israel	US10549052
OptiNoseTM (insufflator)	Nasal spray	Liquid (solution) and powder	Optinose, Yardley, PA, USA	US14167928
Naltos	Nasal spray	Powder	Alchemy, St Mary’s Mill, UK	US10492013
ArcherFish	Nasal spray	Liquid (solution) and powder	Mystic, Seattle, WA, USA	US13770861
Spravato	Nasal spray	Liquid (solution) and powder	Janssen, Titusville, NJ, USA	US16675780 US18023637

**Table 10:** Topic evolutionary type and description.

Evolutionary Type	Description
Newborn	Emerging themes that have only low or no correlation with themes from earlier time windows
Survive	Theme attention is in a stable trend
Split	New themes derived from existing themes that have high correlation but are not very similar to the current theme
Merge	Converging themes that have some correlation with multiple antecedent themes and are the result of multiple themes converging together
Shrink	Theme attention is downwardly trending
Dissolve	Themes generated in subsequent time windows that have no relevance, or very low relevance, to any of the existing themes

**Table 11:** Topics and keywords of N2BD from 1990 to 2023 in different time windows.

Time Window	Topics and Keywords
1990–1994	1_T0: Steroids Administered Nasally to the Hypothalamus 1_T1: Compounds Acting on the Hypothalamus
1995–1999	2_T0: Intranasal Administration of Steroids 2_T1: Calcitonin, Polymer 2_T2: Intranasal Administration of Vitamin B12 2_T3: CNS and Migraine Headache 2_T4: CNS and Vitamins 2_T5: Nasal Administration of Progesterone to the Hypothalamus 2_T6: Polyvinyl Alcohol and Growth Factors
2000–2004	3_T0: Vitamin B12 and Acetylcholinesterase Inhibitors 3_T1: Migraine Alzheimer’s 3_T2: CNS, Parkinson’s Disease, Hypothalamus 3_T3: Transnasal Therapeutic Agents for Migraine Headaches 3_T4: Meningitis Inhibitor Kit (Transnasal) 3_T5: Herbal Medicine Transnasal Treatment for Ischemic Brain Disease 3_T6: Nasal Spray, Hyperosmolar Sugar Composition, Plant Essential Oil 3_T7: Apomorphine Combination Nasal Delivery Formulation 3_T8: CNS Naloxone Nasal Administration 3_T9: CNS and Dopamine Agonists 3_T10: Meningitis and Mucosal Vaccines 3_T11: Nalmefene Hydrochloride Nasal Delivery Formulation 3_T12: DELTA-9-Tetrahydrocannabinol (THC) Delivery
2005–2009	4_T0: ACE Inhibitors Blood–Brain Barrier 4_T1: Sialic Acid, Rhepo 4_T2: CNS, Peptides, Obesity 4_T3: CNS, Macromolecules, Iron Chelator Deferoxamine (DFO) 4_T4: Vitamin B12, Blood–Cerebrospinal Fluid Barrier 4_T5:Aerosol, Herbal Rhinitis, Clenbuterol
2010–2014	5_T0: Nasal Gel, Poloxamer, CNS 5_T1: Growth Factors Lysosomes 5_T2: CNS, Liposomes, Nasal Powder 5_T3: Drug Delivery Devices, Olfactory Zone, Propellants 5_T4: Sem grafts, Depression, Parkinson’s Disease 5_T5: Trigeminal Nerve, Botox 5_T6: Antibiotics, Brain Injury 5_T7: Vitamin B12, CSF 5_T8: CNS, NSAIDs, Alzheimer’s disease 5_T9: Polymer Nanoparticles
2015–2019	6_T0: Disposable Transnasal Brain-Targeted Drug Delivery Device 6_T1: Nasal Spray for Neurodegenerative Diseases 6_T2: Chitosan, Stem Cells, Alzheimer’s Disease 6_T3: CNS, Gene Therapy, Nanotechnology 6_T4: CNS, Puerarin, AD, Peptides 6_T5: CNS, Liposomes, Poloxamer 6_T6: Intranasal Delivery of Glutamate Carboxypeptidase (GCP-II) Inhibitors 6_T7: Traumatic Brain Injury, Peptide-Mimicking Calpain Inhibitors 6_T8: Polymer Nanoparticle Drug Delivery System 6_T9: Transnasal Microemulsions 6_T10: Stem Cells
2020–2023	7_T0: Alzheimer, Dantrolene 7_T1: Exosomes, Stem Cells, Nanoscale 7_T2: Calamus, Blood–Brain Barrier, Neurons 7_T3: Stem Cell, CNS, mRNA 7_T4: COVID-19, Respiratory System, Coronavirus 7_T5: N-Acetyl Cysteine (NAC), Brain Injury 7_T6: Polymers, Liposomes, Aromatic Dialdehydes 7_T7: Brain Injury, Polymers, Nanostructured Lipid Carriers (NLC) 7_T8: Modified Emulsion, Nasogel 7_T9: CNS, Olfactory Region Delivery 7_T10: Antidepressant, Nasal mucosa, Liposome 7_T11: Chitosan Nanocomplexes

## Data Availability

The original contributions presented in the study are included in the article, further inquiries can be directed to the corresponding author/s.

## Abbreviations

BBB	Blood–brain barrier
CNS	Central nervous system
N2BD	Nose-to-brain delivery
CMO	Contract Research Organization
CDMO	Contract Development and Manufacturing Organization
R&D	Research and development
FDA	Food and Drug Administration
NMPA	National Medical Products Administration
EMA	European Medicines Agency
PDMA	Pharmaceuticals and Medical Devices Agency
IPC	International Patent Classification
CPC	Cooperative Patent Classification
LDA	Latent Dirichlet Allocation
TIAB	Title and/or abstract
No.	Number
